# The Effect of a Scanning Strategy on the Residual Stress of 316L Steel Parts Fabricated by Selective Laser Melting (SLM)

**DOI:** 10.3390/ma11101821

**Published:** 2018-09-25

**Authors:** Di Wang, Shibiao Wu, Yongqiang Yang, Wenhao Dou, Shishi Deng, Zhi Wang, Sheng Li

**Affiliations:** 1 Department of Mechatronics Engineering, School of Mechanical and Automotive Engineering, South China University of Technology, Guangzhou 510640, China; mewdlaser@scut.edu.cn (D.W.); siberghost@126.com (S.W.); dwh68@outlook.com (W.D.); shishidengscut@163.com (S.D.); 2 School of Metallurgy and Materials, The University of Birmingham, Birmingham B152TT, UK; s.li.2@bham.ac.uk

**Keywords:** selective laser melting, divisional scanning, residual stress, deformation

## Abstract

The laser scanning strategy has an important influence on the surface quality, residual stress, and deformation of the molten metal (deformation behavior). A divisional scanning strategy is an effective means used to reduce the internal stress of the selective laser melting (SLM) metal part. In order to understand and optimize the divisional scanning strategy, three divisional scanning strategies and an S-shaped orthogonal scanning strategy are used to produce 316L steel parts in this study. The influence of scanning strategy on the produced parts is verified from the aspects of densification, residual stress distribution and deformation. Experiments show that the 316L steel alloy parts adopted spiral divisional scanning strategy can not only obtain the densification of 99.37%, but they also effectively improve the distribution of residual stress and control the deformation degree of the produced parts. Among them, the spiral divisional scanning sample has the smallest residual stress in plane direction, and its σ*_x_* and σ*_y_* stress are controlled within 204 MPa and 103 MPa. The above results show that the spiral divisional scanning is the most conducive strategy to obtain higher residual stress performance of SLM 316L steel parts.

## 1. Introduction

Selective laser melting technology adopts single-point high-energy laser beam to melt metal powder layer by layer along the filling path of three-dimensional discrete profile. Theoretically, it is not constrained by the structure of the part and can fabricated any complicated structural part with the densification of nearly 100% [1,2,3,4]. In fact, many factors affect the performance of selective laser melting (SLM) parts, not only by the process parameters including laser power, scanning speed, scanning space, layer thickness, and spot size et al. [5,6,7,8], but also by the laser scanning strategy and the length of the scanning line [9].

The strategy planning software automatically generates the scanning lines according to the selected filling algorithm, based on the discrete slice data of the three-dimensional model of the produced part. The choice of scanning strategy is very important to the quality improvement of SLM parts [10]. The scanning strategy has a great influence on the heat distribution of the part and plays a decisive role in the residual stress distribution and the deformation trend of the part [11]. In a microscopic view, the scanning strategy affects the grain growth direction and grain size, and it determines the microstructure of the part [12]. The preferred scanning strategy usually controls the length of the scanning lines. Excessive scanning lines can not only decrease the stability of the melt track, but they also reduce the surface quality of the parts. The residual stress can also accumulate in a single direction, and affect the residual stress distribution.

Many scholars conducted a series of studies on the influence of scanning strategy on the performance of SLM parts. These scholars initially studied some simple scanning strategies and adopted finite element simulation methods to study the impact on the performance of parts. Kruth et al. [13] adopted a sub-block scanning method by using a rectangular block of equal length and width and found that the previous scanning region could preheat the adjacent region well and decrease the temperature gradient. Kruth et al. [14] also designed a “bridge curvature method” to study the influence of the scanning line’s length, scanning line direction and island scanning strategy on residual stress. It is found that the short scanning line and the optimal orientation of scanning line can effectively reduce the residual stress. Parry et al. [15] simulated the temperature field and the stress field in Z-shaped and S-shaped scanning mode by finite element method and studied the effect of scanning line length and scanning area on temperature field and stress field. It is suggested that too long scanning lines should be avoided during SLM process. With the development of SLM technology, many researchers began to carry out more detailed research on scanning strategies. Lu et al. [16] studied the influence of the size of island shape on the densification, microstructure, and mechanical properties and residual stress of the part, and the optimum width of the region was found to be within 5 mm to 7 mm. Thijs et al. [17] studied the microstructure evolution of SLM parts based on three scanning strategies: zigzag scanning, unidirectional scanning, and orthogonal scanning. Qian et al. [18] studied the residual stress and deformation of SLM parts in helix scanning strategy. Beal et al. [19] used different scanning strategies to statistically analyze the effects with respect to the composition of Cu, and found that the refill strategy produced better results compared to the other scanning strategies. As for the divisional scanning method, Yasa et al. [20] also explored the influence of divisional scanning strategy on densification, surface quality, mechanical properties, and residual stresses formed during SLM, and it was concluded that divisional scanning had some advantages such as lower residual stresses and better surface quality. Rashid et al. [21] also discussed the effect of scan strategy on densification and metallurgical properties of 17-4PH parts fabricated by SLM, and he found that the samples fabricated with double scanning strategy showed an improvement in densification, as compared to that fabricated with a single scanning strategy. Geiger et al. [22] tailored the texture of IN738LC as processed by SLM with specific scanning strategies, and they found that the applied laser scanning strategies allowed the crystallographic texture to be tailored locally.

Many scholars above have studied the influence of the scanning strategy on the quality of SLM parts based on Z-shaped scan, S-shaped scan and divisional scan, and put forward several kinds of divisional scanning methods according to specific problems. However, the effect of different divisional scanning methods on the performance of SLM parts is still not specific. Currently, the mainstream SLM equipment vendors such as Concept Laser, SLM Solutions, and EOS have adopted the divisional scanning strategy on their commercial SLM machines. However, the divisional scanning strategies adopted are all different with each other, and further research on the different divisional scanning strategy is still scarce. Therefore, on the basis of the aforementioned researchers, three kinds of divisional scanning strategies are developed to study the influence on the densification, surface quality, residual stress distribution and deformation of SLM parts in this paper.

## 2. Research Methods

### 2.1. Experimental Equipment and Materials

**Figure 1:**
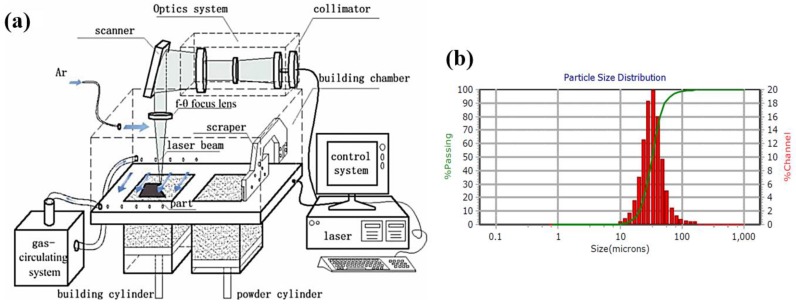
(**a**) Principle of selective laser melting (SLM) manufacturing equipment; (**b**) particle size distribution (PSD).

The experiment was carried out using Dimetal-100 SLM equipment, which was developed independently by South China University of Technology, Guangzhou, China, as shown in Figure 1. The equipment is mainly composed of a fiber laser, optical transmission regulation system, gas circulation system, fabricating room, laying powder device, cooling system, and the main control software. The laser was guided by a high-precision optical path transmission system, and it was finally focused by the f-θ lens (f is the focal length) to ensure that the power density of the spot was basically the same throughout the working plane. The maximum fabricating size of the equipment is 100 mm × 100 mm × 100 mm, the scanning speed is 10–7000 mm/s, the focused spot diameter is 70 μm, and the precision can reach ± 0.1 mm. The SLM process was protected by an argon atmosphere (<500 ppm of oxygen), and the purity of argon gas was 99.999%. 316L stainless steel is a common austenitic steel with excellent corrosion resistance, which plays an important role in many fields. Also, SLM has many advantages over traditional machining methods, such as geometric freedom, personalization, and better mechanical properties. Thus, 316L steel was chosen appropriately for the fabricated materials in the study of SLM residual stress. The experimental material was 316L steel, which has a good fluidity, with an average particle size of 30.32 μm. The chemical composition of 316L steel powder is shown in Table 1. In order to ensure the processing quality of produced parts, the optimized process parameters were selected, as shown in Table 2.

### 2.2. Experimental Method

**Figure 2:**
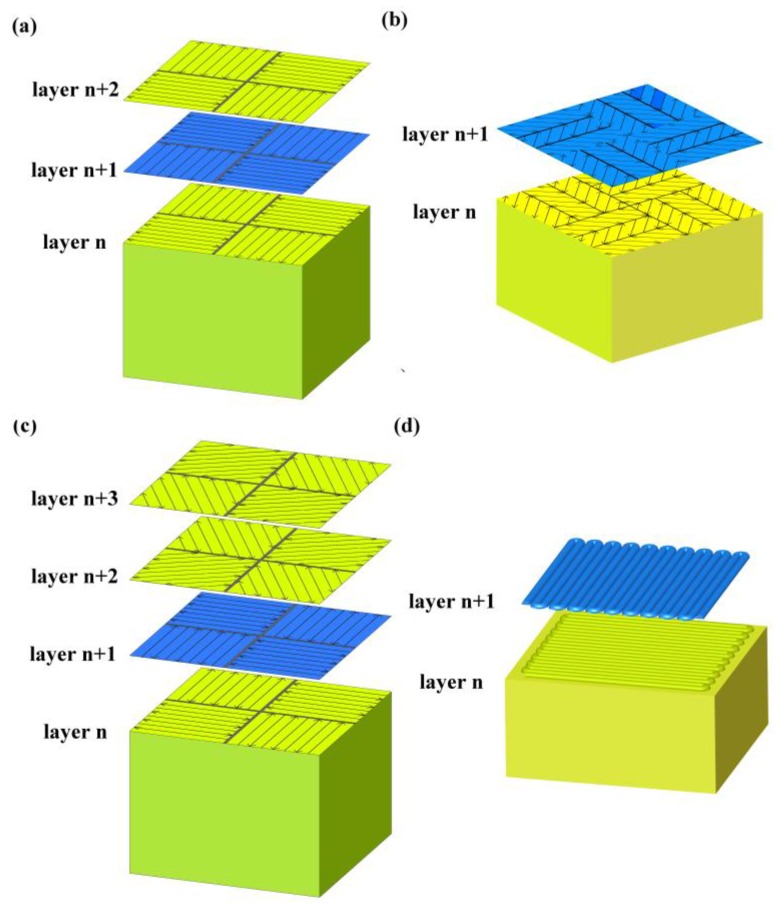
Schematic of three different divisional refilling strategy and “s” style orthogonal scanning strategy: (**a**) normal partition strategy; (**b**) oblique line and layer-staggered divisional strategy; (**c**) spiral divisional strategy; (**d**) “s” style orthogonal scanning strategy.

Three kinds of self-developed divisional scanning strategies, normal partition strategy, oblique line, and layer-staggered divisional strategy and spiral divisional strategy, were used in the experiment for the study of residual stress in SLM. At the same time, the commonly used “s” style orthogonal scanning strategy was introduced as a contrast reference for the above-mentioned three divisional scanning strategies. Among them, the “s” style orthogonal scanning strategy is a scanning strategy where the cross section is filled with S-shaped scanning lines, and the upper and lower scanning lines are staggered. The scanning lines of the upper two layers are orthogonal to the x and y directions of the lower two layers, as shown in Figure 2d below. Significantly, the S-shaped scanning lines refer to the mode of the adjacent scanning line; when a scanning line is finished, the origin of the next adjacent scanning line will be next to the destination of the finished scanning line.

A normal partition strategy is achieved by dividing the section into the isometrical square sub-areas, and allowing a small amount of overlap between the each two adjacent square areas in the same layer. The overlap area is generally set to equal as a spot diameter. The S-shaped scanning lines follow the rules that the scanning lines in adjacent areas are orthogonal, and that the scanning lines belonging to the same area in the upper and lower layers are orthogonal, as shown in Figure 2a.

A division nested mechanism was applied for the design of oblique lines, and the layer-staggered divisional strategy. In this strategy, the adjacent layers’ divisions were orthogonally filled with three parallel rectangular sub-regions respectively. And the adjacent x, y direction division regions are filled with oblique 45° and 135° scanning line. This strategy can not only reduce the number of the areas, but it can also limit the length of the scanning lines inside each area at the same profile shape. This strategy’s scanning lines follow the rules as shown in Figure 2b.

The spiral divisional strategy adopted the divisional direction rotation mechanism. Every two adjacent layers are called a group, and the direction between every two adjacent groups is set to 60°. Besides, the S-shaped scanning lines that belong to the same sub-area in different layers of the same group should be orthogonal, and the scanning lines in the adjacent sub-areas are also orthogonal, as illustrated in Figure 2c.

In order to reduce the negative impact of the width of region segmentation on the part performance, reasonable division size parameters should be chosen. When the width of the sub-area is too small, the area connection is unstable and prone to produce micro-pores. Also, the same cross-section needs to be divided into more areas, and more area connections are introduced, which may affect the densification of the parts. When the sub-area width is far too large, the length of the scanning line is too long, resulting in large residual stress inside the part. The width of the sub-area is suitable for the choice of 5 mm–7 mm [17]. Therefore, the area width in this paper was selected as 5 mm. The animated videos about those divisional strategies are available in the Supplementary Materials.

### 2.3. Test Methods

#### 2.3.1. Densification Detection and Surface Quality Analysis

Four groups of 10 mm ×10 mm × 10 mm cubic samples were experimentally fabricated, and each group consisted of three parts which were group #1 (normal partition strategy), group #2 (oblique line and layer-staggered divisional strategy), group #3 (spiral divisional strategy), and group #4 (“s” style orthogonal scanning strategy). The densification of the SLM parts was measured by Archimedes principle. The weights of the samples in air and water were measured successively, and the calculation formula of the drainage method is as in Equation (1):

(1) $$\rho =\left(\frac{W_{\mathrm{air}}\times \rho_{H_{2}O}}{W_{\mathrm{air}}-W_{H_{2}O}}\right)/\rho_{0},$$

where in $\rho_{H_{2}O}$ = 1.00 g/cm^3^ is the density of distilled water, *W*_air_ is the average weight of the fabricated parts in the air, $W_{H_{2}O}$ is the average weight of the fabricated parts in water, and *ρ*_0_ = 7.98 g/cm^3^ is the theoretical density of 316L steel alloy.

The first three groups of the cubic samples did not undergo any surface treatment, and the overall surface quality of each group and the surface area at the junction quality were observed using the VHX-5000 (KEYENCE company, Osaka, Japan) at a magnification of 20 and 200 times.

#### 2.3.2. Measurement of Residual Stress Distribution and Deformation

**Figure 3:**
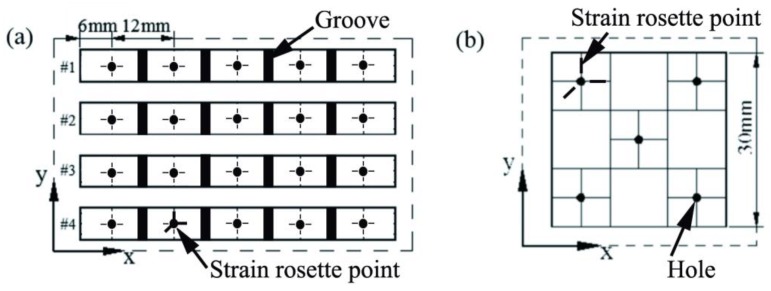
Design model of residual stress testing: (**a**) Residual stress in the direction of length; (**b**) residual stress in the plane distribution.

On the base of relevant references [23], is clear that the accumulated heat in the fabricating part is gradually uniform with an increase of fabrication height. Therefore, the subsequent laser energy input would temper the fabricated layer and release the stress. The residual stress that we discussed in this article was biased toward the distribution in horizontal direction. Thus, two geometric shape samples were designed to test the influence of the scanning strategy on the residual stress: (1) strip samples with a width of 6 mm, a thickness of 10 mm, and a length of 60 mm; (2) square samples with a width and length of 30 mm, and a thickness of 7 mm. Based on those samples, the influence of the different division scanning methods on the stress distribution in the longitudinal direction, and the overall plane stress distribution of SLM parts was discussed. For the two kinds of geometric models, four kinds of samples were processed respectively in group #1 (normal partition strategy), group #2 (oblique line and layer-staggered divisional strategy), group #3 (spiral divisional strategy), and group #4 (“s” style orthogonal scanning strategy). According to ASTM E837, the residual stress of the produced parts is measured by a drilling method. Generally, the drilling method can measure residual stress with a measurement accuracy of 20%. The satisfactory residual stresses in the fabricated parts can be controlled to within 60% of the yield stress. The distribution of the residual stress test points of the strip and square samples are shown in Figure 3a,b. In Figure 3a, the strip sample was designed to discuss the stress distribution in a longitudinal direction with a groove and the typical three-element clockwise stress rosette. The aim of the groove, with a depth of approximately 2 mm, was to relieve residual stress. The holes in Figure 3b were also used to relieve stress, but the difference was that the three-element stress rosette was set as shown in Figure 3b to discuss the overall plane stress distribution of SLM parts. The diameter and depth of hole are 5 mm and 6 mm, respectively.

**Figure 4:**
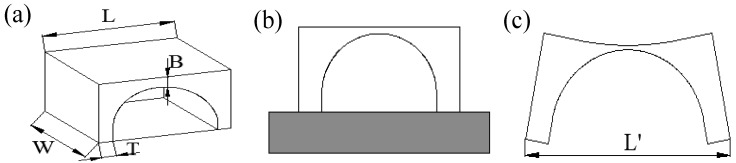
Schematic of bridge structure designing: (**a**) 3D design model of the part; (**b**) placement and manufacturing of parts; (**c**) schematic of parts deformation.

In addition, the SLM parts were cut from the substrate. The parts were warped upwards at both ends, and they formed a certain arc in the middle due to residual stress. According to the trends and characteristics of this deformation, an arch-like bridge structure was designed. The arch structure is as shown in Figure 4, and the dimensions of the retaining structure *W*, *T* and *B* are constant, with *W* = 10 mm, *T* = 2 mm and *B* = 1 mm. The size *L* was set to 12 mm, 20 mm, or 28 mm. These three gradients were used to verify the influences of different lengths and scanning methods on the deformation of the parts. For each designated length, four groups of samples were fabricated, which were group #1 (normal partition strategy), group #2 (oblique line and layer-staggered divisional strategy), group #3 (spiral divisional strategy), and group #4 (“s” style orthogonal scanning strategy). According to the deformation characteristics of the specimen, the sample deforms, as shown in Figure 4b, under the joint action of the internal residual stress in a multi-direction. After measuring the distance *L* of the outer edge of the bottom of the bridge structure, as shown in Figure 4b, the deformation of the bridge structure was measured by *δ* = *L*’ − *L*. The deformation of each sample is measured in three positions, and the mean value is the result of measurement.

## 3. Results and Discussion

### 3.1. Densification and Surface Quality Analysis

**Figure 5:**
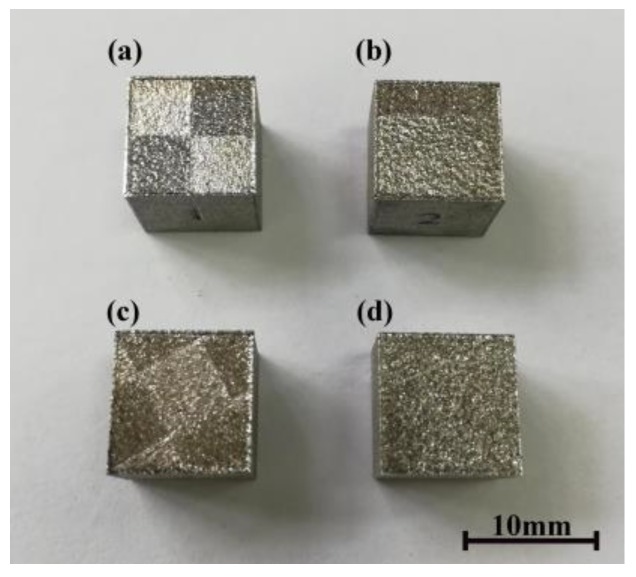
Testing sample of densification: (**a**) normal partition strategy; (**b**) oblique line and layer-staggered divisional strategy; (**c**) spiral divisional strategy; (**d**) “s”-style orthogonal scanning strategy.

Figure 5 shows the fabricated cubic samples. The density of the samples was calculated by the Archimedes principle. The average densities of the (a), (b), (c) and (d) groups were 99.28%, 99.37%, 99.10%, and 98.64% respectively. No big differences existed among the four groups of samples. The density of the produced parts adopting a divisional scanning strategy was up to 99%, which indicates that the parts adopting three kinds of divisional scanning strategies could obtain a dense sample.

**Figure 6:**
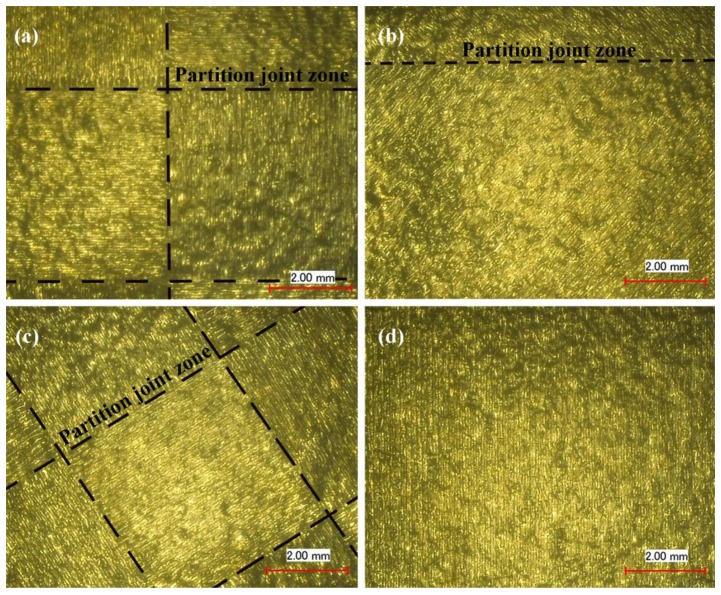
The surface morphology of samples (magnification 20×): (**a**) Normal partition strategy; (**b**) Oblique line and layer-staggered divisional strategy; (**c**) Spiral divisional strategy; (**d**) “s” style orthogonal scanning strategy.

Figure 6 illustrates the surface morphology of the first three groups of the square blocks at 20 times magnification. As can be seen from the surface morphology in Figure 6, although there were some small holes in the surfaces of the three group samples, the overall surfaces were uniform. The fusion tracks were coherent and clear with a good overlap effect, and the overlap areas between the different zones in the three group samples were also good.

**Figure 7:**
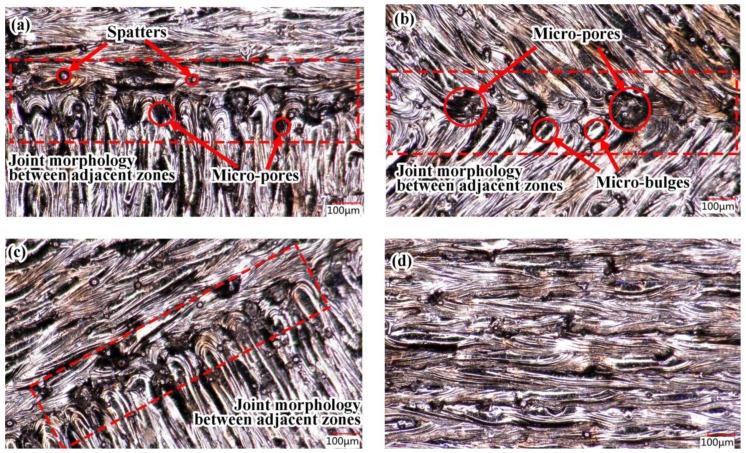
The surface morphology graph of overlap (magnification 200×): (**a**) Normal partition strategy; (**b**) Oblique line and layer-staggered divisional strategy; (**c**) Spiral divisional strategy; (**d**) “s” style orthogonal scanning strategy.

**Figure 8:**
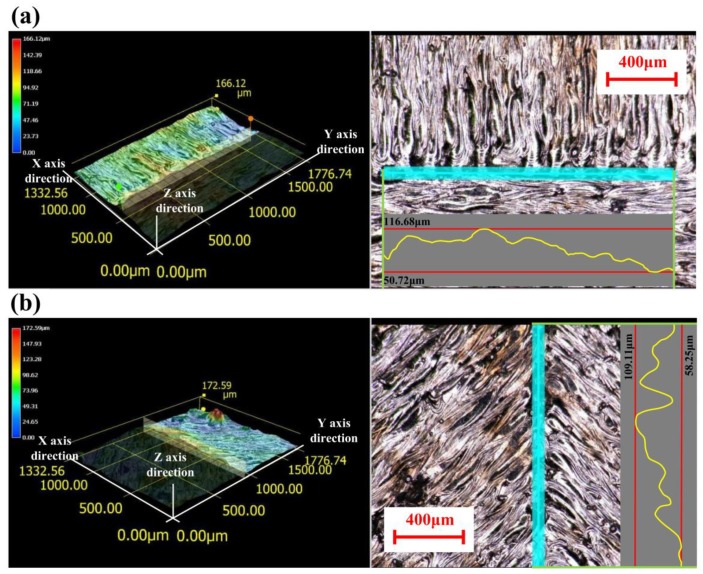
Surface measurement of overlap: (**a**) Overlap between two groups of scanning lines which was used in the normal partition strategy and the spiral divisional strategy; (**b**) Overlap between a single scanning line and one group of scanning lines, which was used in the oblique line and layer-staggered divisional strategy.

Figure 7 illustrates the morphologies of the overlap area of the first three group blocks at a magnification of 200 times. The results shows that there were obvious microscopic defects, such as micro-pores and micro-bulges. Figure 8 shows the surface measurement of overlap, including the 3D topography of the overlap between two groups of scanning lines, and the overlap between a single scanning line and one group of scanning lines. The Blue line represents the range of measurement, the overlap region. The curves are the measured values of the heights of the overlapped regions. According to the curves from Figure 8a,b, in the oblique line and layer-staggered divisional strategies, there were periodic micro-pores and micro-bulges existing on the surface along the overlap area, with Rz = 40.9 μm. Due to the primitive overlap of the origins of the scanning line, the surface quality of the overlap area was relative poor. The overlap between two groups of scanning lines had a smaller Rz, with 36.8 μm. It could be seen from Figure 7a,c that there was a small amount of micro-pores and micro-bulges in the overlap area of sample groups #1 and #3, but this did not affect the overlapping, and the curve was smoother. It reveals that the micro-pores in sample groups #1 and 3 were shallower than the pores, with a depth of approximately 20 μm in group #2 samples.

**Figure 9:**
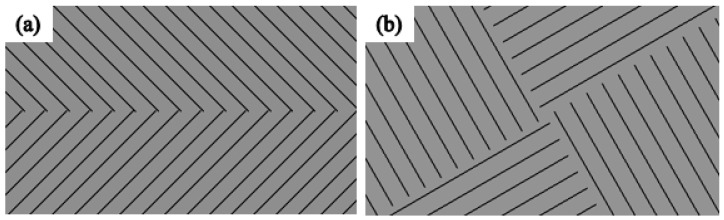
Mechanism of track overlap: (**a**) Overlap between two groups of scanning lines; (**b**) overlap between a single scanning line and one group of scanning lines.

The above results show that the adjacent region using a rectangular overlapping mechanism and a spiral divisional mechanism can achieve a better regional overlapping effect. The overlapping effects with one scanning line in the adjacent area and multiple scanning lines in the other area (Figure 9b) were better than another overlapping with multiple scanning lines in the adjacent area. The reason is that in the overlapping mode of multiple Figure 9b, the micro-pores between the semi-circular origin of the parallel track were filled first, or later by a single vertical track. Technically, the micro-pores or even the micro-holes deriving from the “Match head” shape, which were influenced by the delay time of laser on and laser off, and the delay time of the galvo scanning system. This was an inevitable technical feature in laser scanning, but a prominent defect in the divisional scanning strategy.

### 3.2. Residual Stress Distribution Analysis

**Figure 10:**
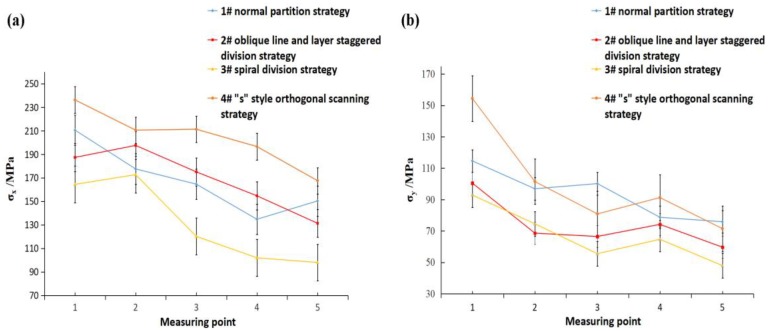
Distribution of residual stress: (**a**) X-direction residual stress distribution curve; (**b**) Y-direction residual stress distribution curve.

Figure 10 shows the average results of the first residual stress test model of four group specimens. It can be seen from Figure 10a that the residual stresses of the four group samples are all at a relatively normal level within the range of 98 MPa to 240 MPa, and the σ_x_ values of all the four samples are larger at the scanning start position and showed a downward trend along the scanning direction. The declining trend of group samples #1, #2 and #3 were more obvious than that of group #4 samples, which was due to the fact that the melting track was surrounded by the powder at the scanning starting position, which thus affected the heat conduction and resulted in greater thermal stress. As the scanning continued forward, the temperature gradient of the melt track tended to be in a state of dynamic equilibrium, and the thermal stress gradually decreased. However, for the group #4 samples, the “s” style orthogonal scanning strategy was used to plan the path, the scanning line was longer, and the scanning direction of each layer was substantially the same. As a result, the thermal stress was accumulated along the scanning direction [24], and the residual stress was large. For #1, #2, and #3 group samples, the divisional scanning method was adopted, the length of the scanning line was set to 5 mm, the control was within the range of 8 mm, and the scanning lines in each adjacent sub-area were orthogonally crossed, reducing the thermal stress accumulation along a single direction, and the stress was decreased along the scanning direction.

Comparing Figure 10a with b, it was shown that the distributions of σ_y_ of samples from groups #1, #2 and #3 along the x-direction were more stable than σ_x_, and they varied from 50 MPa to 115 MPa. The distribution of σ_y_ of group #4 samples along the length was similar to σ_x_. This is because each sub-area scan fabricated by divisional scan strategy planning were all scanned along the x-direction, resulting in a temperature gradient in the x-direction that was greater than the y-direction. Therefore, the residual stress in the x-direction was greater than that in the y-direction [15]. For the “s” style orthogonal scanning strategy, the upper two layers were orthogonal to the x and y directions of the lower two scanning lines, so that the temperature gradient distribution in the x direction and the y direction were almost similar.

**Figure 11:**
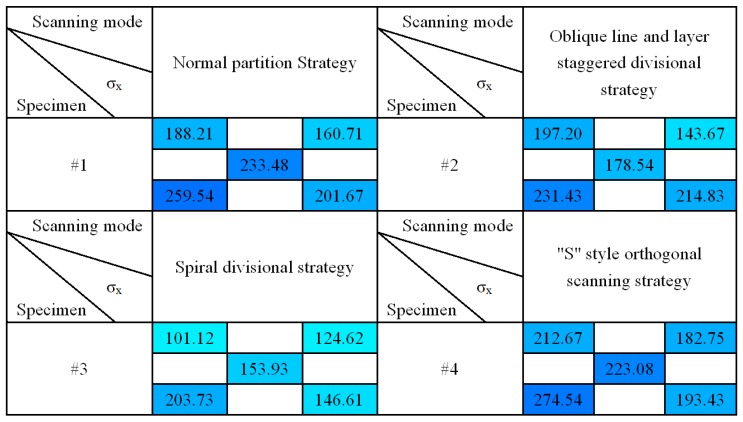
The result of X-direction residual stress σ*_x_*.

**Figure 12:**
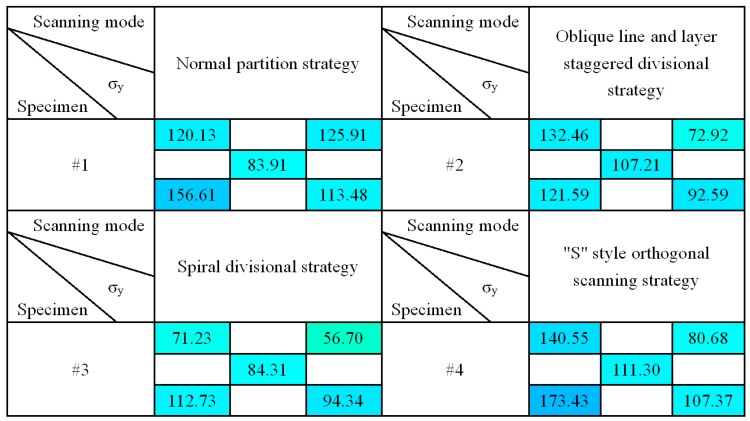
The result of Y-direction residual stress σ*_y_*.

Figure 11 and Figure 12 show the results of group #2 of four stress test models, σ_x_ and σ_y_. As can be seen from the table, for the four samples, σ_x_ values were larger in the lower left corner of the sample, and the stress showed a decreasing trend upward and right, which was due to the scanning start position being located in the left lower corner of the fabricating plane, following the scanning sequence from bottom to top, the left lower part of the produced part presents a larger temperature gradient field, and then it tends to be in a state of dynamic equilibrium. It was also found that the overall stress values of the group #2 and #3 samples were smaller, and the distribution was more uniform. The stress values of the groups #1 and #4 samples were larger, which was due to the normal partition strategy adopted on group #1 samples; the sub-area width of each layer was 5 mm, and the overlapping area between the areas was re-melted, which increased the input energy of the overlapping area, resulting in the accumulation of large amounts of thermal stress in the overlapping area. In the group #4 samples with an “s” style orthogonal scanning strategy, the scanning line was much longer, which could easily intensify the stress accumulation effect. The result showed that the staggered divisional strategy and spiral divisional strategy could effectively reduce the residual stress in the profile of the produced parts and improve the distribution of residual stress to make the residual stress evenly distributed.

Overall, most of the measured results were a little bit less than the residual stress value when 316L SLM parts adopt single-direction scanning strategy or other simple scanning strategies [15,23]. Actually, the SLM parts’ residual stress relates to many factors, such as materials, part height, processing parameters, and even processing equipment. However, based on the existing related literature [15,23], Figure 10, Figure 11 and Figure 12 do not just show the distribution characteristics of the residual stress in a longitudinal direction, but they also reveal the tendency effect of the scanning line’s direction and length to the residual stress, with three complicated divisional strategies.

### 3.3. Deformation Analysis

**Figure 13:**
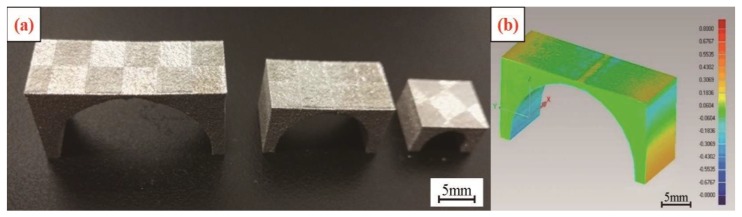
The structure and deformation diagram of bridge samples fabricated by a spiral divisional strategy: (**a**) A sample of a bridge-shaped structure; (**b**) the deformation diagram.

Figure 13a shows a sample of a bridge-shaped structure fabricated by a group #3 scanning strategy (without heat treatment). After the sample was fabricated, a reverse engineering method was used to obtain the actual 3D model of the samples. The theoretical model and the reconstructed model of the sample were then compared and analyzed in Geomagic software, and the deformation diagram of the SLM part was obtained. According to the schematic diagram 13b, it could be seen that the bottom of the bridge structure was obviously yellow, and it appeared to be warped out. The tip of the bridge shaped pillar was obviously blue, and internal contraction appeared. The top beam of the bridge-shaped part also warped up at both ends, while the middle part of the beam was compressed.

**Figure 14:**
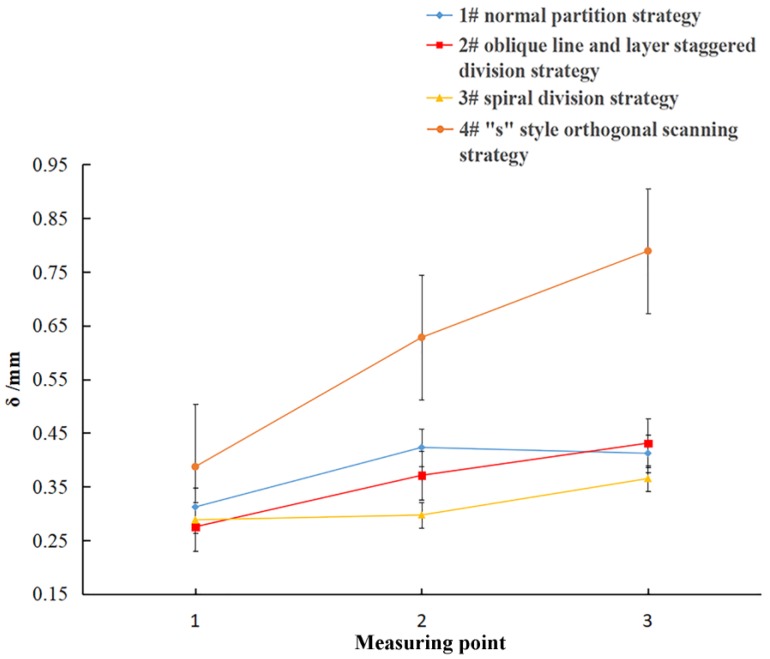
Relationship between the deformation and scanning line lengths under different scanning strategies.

As shown in Figure 14, this was the deformation result of the of the four group samples. The curves of samples from groups #1, #2 and #3 group samples in the figure changed gently. With the length of the bridge structure increased from 12 mm to 28 mm, and there was a slight change in the deformation, with a difference of only 0.2 mm. There is no obvious difference among the deformations of the three groups samples, which showed that the deformation of the sample using the divisional scanning strategy had little to do with the length of the bridge structure, and the amount of deformation did not change significantly with the length of the bridge structure. However, the deformation of group #4 samples obviously increased with the length of the bridge, and the deformation of each sample in group #4 was greater than the deformation of the first three groups of samples. When the length was 12 mm, the difference of the deformation was only 0.1 mm. When the length was 28 mm, the difference of the deformation increased to 0.4 mm. Obviously, with a length increase in the bridge structure, the deformation difference between group #4 samples and the first three sample groups became larger and more obvious.

The reason for the above result is related to the length of the scanning line. The first three kinds of divisional scanning strategies all defined area lengths of 5 mm, and the divisional scanning strategy used a sub-area boundary to cut off the scanning lines, so that the scanning line length was controlled to within 8 mm. Therefore, when the bridge structure length becomes longer, the scanning line’s lengths in these three kinds of divisional scanning strategies does not increase, and the deformation will not increase. All of the divisional scanning strategies use orthogonal scanning lines in the adjacent sub-areas, so that the deformation in the same direction will not have a cumulative effect along the length of the bridge structure, and the strategies also control the deformation of samples. However, when the “s” style orthogonal scanning strategy is applied to generate the scanning lines, the length of the scanning line is closely related to the size of the sample model. As the size of the model increases, the length of the scanning line and the deformation will also increase.

Therefore, the scanning strategy works by indirectly changing the length and direction of the scanning lines, so that the length of the scanning line is not limited by the size and shape of the three-dimensional model. Besides, the deformation of the produced parts can be improved by controlling the length of the scanning lines to within a certain range, and changing the direction of the scanning lines along the length direction. Therefore, it is important to choose the appropriate scanning strategy to control the deformation of the SLM parts.

### 3.4. Discussions

**Figure 15:**
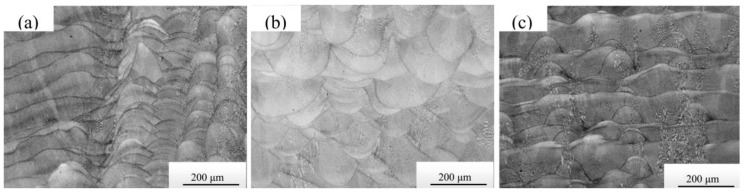
Microstructures of three different scanning strategies: (**a**) Normal partition strategy; (**b**) spiral divisional strategy; (**c**) “s” style orthogonal scanning strategy.

The experimental results show that all three kinds of divisional scanning strategies can obtain dense parts with a density exceeding 99%. As for the overlap area, the intersection of the slash layer-staggered divisional scanning area, where multiple scanning lines in one area are abutted with multiple scanning lines in another area, is prone to the phenomenon of insufficient powder compensation and micro-pores, which are very unfavorable for the improvement of the overlap quality. Because normal partition strategy adopts a scanning line outward extension mechanism to overlap, the overlap joint areas are re-melted. Thus, the energy input is too large in the overlap of the joint areas, resulting in local excessive residual stress and affecting the residual stress distribution uniformly in the fabricated plane. However, the residual stress of the part plane with an oblique line and layer-staggered divisional strategy and spiral divisional strategy are all at a low level, and the distribution is more uniform. All three kinds of divisional scanning strategies can limit the length of the scanning line to 8 mm, so that the deformation of the part is not affected by the size of the part, and the effects of the three kinds of divisional scanning on the part deformation are not significantly different. The above results might provide a reference for optimizing the scanning strategy of selective laser melting. For example, when using the divisional scanning strategy, the scanning mechanism of docking the scanning lines in the adjacent sub-area should not be adopted, as it will affect the surface quality. Minimizing the size of the in-plane scanning partition, helps to distribute the residual stress evenly and to keep it at a low level. In fact, for the defects in overlap, Yasa et al. also found that the selection of parameters related to sectoral scanning may cause aligned porosity at the edges between sectors or scanned tracks, which is very undesirable in terms of mechanical properties [20]. The authors also made a microscopic analysis on the overlap of the melting track. As shown in Figure 15, it can be found that the microstructure of the samples using three scanning strategies have obvious different track overlapping characterizations.

The above analysis shows that the divisional scanning strategy can inevitably encounter regional overlap problems, and the quality control of overlap area has a crucial influence on the mechanical properties and surface quality of the parts. For example, Dai et al. investigated the influence of the re-melting behavior and scanning strategy on the formation of the “track–track” and “layer–layer” molten pool boundaries (MPBs) [25], and Almangour et al. investigated scanning strategies for texture and anisotropy tailoring during selective laser melting of TiC/316L stainless steel nanocomposites [10]. Carter et al. from Birmingham University also observed the repeating pattern shown in the grain structure, which has been linked to the overlapping of the ‘island’ pattern used, as is standard in the Concept Laser M2, and they suggested that the formation of this bi-modal grain structure can be linked to heat transfer away from the solidifying melt pool [26].

Of course, the design of this experiment is not well considered. For example, only the three divisional scanning strategies developed based on the current mainstream laser scanning strategies were selected to investigate the influence of the division mechanism on the performance of the SLM part, and other division mechanisms were not fully introduced. The drilling method was adopted to test the residual stress. Due to the constraints of the strain force, the selected test points were limited and the interval was large, and this could only reflect the general rules of residual stress distribution. At the same time, the effects of different divisional scanning strategies on the performance of SLM parts were evaluated only from the aspects of densification, surface quality, residual stress distribution, and deformation. The effect of different divisional scanning modes has not been further explored within the microstructure of parts, and a follow-up remains to be further studied.

## 4. Conclusions

According to the three kinds of self-developed divisional scanning strategies, the influence of different divisional scanning strategies on the performance of SLM fabricated parts is discussed from the aspects of densification, surface quality, residual stress distribution, and deformation. The main conclusions of this work are as follows:

1. It was found that by using the normal partition strategy, the SLM parts had a large amount of residual stress in the overlap area, which was not conducive to residual stress distribution in the fabricated plane. Using an oblique line and layer-staggered divisional strategy easily forms micro-pores and affects the surface quality of parts, but the overlap regions in the adjacent layers are not located in the same vertical plane, so that the residual stress distribution of the components is more uniform, and the value is smaller.
2. Among the three kinds of divisional scanning strategies, the spiral divisional strategy is the best for obtaining produced parts with better performance. 316L steel alloy parts adopted a spiral divisional scanning strategy, and this not only obtains a density of 99.37%, but it also effectively improves the distribution of residual stress and controls the deformation degree of the produced parts.
3. The influence of scanning strategy on the deformation of the SLM-produced part is achieved by indirectly changing the length and direction of the scanning lines. The deformation of the produced part can be improved by controlling the length and direction of the scanning lines.

## Figures and Tables

**Table 1:** Powder composition of 316L (mass fraction, wt %).

Element	Content (%)
C	0.03
Cr	17.5
Ni	12.06
Mo	2.06
Si	0.86
Mn	0.3
O	0.09
S	0.032
P	0.029
Fe	Bal.

**Table 2:** SLM processing parameters of 316L steel powder.

**Laser Power/W**	160
**Scanning Speed/(mm/s)**	600
**Layer Thickness/mm**	0.03
**Hatch Space/mm**	0.07
**Spot Compensation/mm**	0.03
**Spot Diameter/μm**	80
**Oxygen Content/%**	≤ 0.01
**Shielding Gas**	Argon

## Supplementary Materials

The following are available online at http://www.mdpi.com/1996-1944/11/10/1821/s1: Video S1: Normal partition strategy DEMO; Video S2: Oblique line and layer-staggered divisional strategy DEMO; Video S3: Spiral divisional strategy.
